# A study of neural-related microRNAs in the developing amphioxus

**DOI:** 10.1186/2041-9139-2-15

**Published:** 2011-07-01

**Authors:** Simona Candiani, Luca Moronti, Davide De Pietri Tonelli, Greta Garbarino, Mario Pestarino

**Affiliations:** 1 University of Genoa, Department of Biology, viale Benedetto XV 5, 16132 Genoa, Italy; 2 The Italian Institute of Technology, Department of Neuroscience and Brain Technologies, Via Morego 30, 16163 Genova, Italy

## Abstract

**Background:**

MicroRNAs are small noncoding RNAs regulating expression of protein coding genes at post-transcriptional level and controlling several biological processes. At present microRNAs have been identified in various metazoans and seem also to be involved in brain development, neuronal differentiation and subtypes specification. An approach to better understand the role of microRNAs in animal gene expression is to determine temporal and tissue-specific expression patterns of microRNAs in different model organisms. Therefore, we have investigated the expression of six neural related microRNAs in amphioxus, an organism having an important phylogenetic position in terms of understanding the origin and evolution of chordates.

**Results:**

In amphioxus, all the microRNAs we examined are expressed in specific regions of the CNS, and some of them are correlated with specific cell types. In addition, miR-7, miR-137 and miR-184 are also expressed in endodermal and mesodermal tissues. Several potential targets expressed in the nervous system of amphioxus have been identified by computational prediction and some of them are coexpressed with one or more miRNAs.

**Conclusion:**

We identified six miRNAs that are expressed in the nervous system of amphioxus in a variety of patterns. miR-124 is found in both differentiating and mature neurons, miR-9 in differentiated neurons, miR-7, miR-137 and miR-184 in restricted CNS regions, and miR-183 in cells of sensory organs. Therefore, such amphioxus miRNAs may play important roles in regional patterning and/or specification of neuronal cell types.

## Background

MicroRNAs (miRNAs) are a large class of non-coding RNAs involved in post-transcriptional regulation. They were first discovered in nematodes and subsequently found to be widely distributed in plants and animals [1-4]. miRNAs are 18 to 24 nucleotides long and regulate translation by binding to the respective mRNA transcripts, either inducing mRNA degradation or directly inhibiting translation. In animals, miRNAs bind to specific bases in the 3' untranslated regions (UTR) of mRNA targets [5]. Each miRNA can regulate translation of several genes and modulate many biological processes such as developmental timing, cell proliferation, cell death, *Hox *gene expression and early embryogenesis [6]. miRNAs play also important roles in stem cell biology, furthermore altered miRNA functions have been implicated in a number of human disorders and cancers [7]. In particular, miRNAs are known to regulate translation of a number of genes involved in development of the central nervous system (CNS) [8-10].

miRNAs are to a large extent evolutionarily conserved. The basal chordate amphioxus has 115 miRNA families, 55 of which are homologous to those of vertebrates [11-13]. Amphioxus has a prototypical chordate genome with a high degree of synteny with vertebrate genomes [14,15], and a body plan that is also similar to that of vertebrates. Although the amphioxus CNS has comparatively few neurons--an estimated 20,000 in the adult [16] and lacks a neural crest [17], the basic organization of the amphioxus CNS is comparable to that in vertebrates. Comparisons of gene expression and mapping of neurons and their connections have indicated that the amphioxus CNS consists of a diencephalic forebrain (the telencephalon is lacking), a small midbrain, a hindbrain and spinal cord [18,19]. The forebrain and small midbrain are together termed the cerebral vesicle. Thus, the most anterior photoreceptor (the frontal eye) is thought to be homologous to the vertebrate paired eyes, the lamellar body in the dorsal part of the amphioxus diencephalon is considered to be homologous to the vertebrate pineal complex, and secretory cells in the floor of the diencephalon are thought equivalent to the vertebrate infundibulum which produces Reissner's fiber [19]. In addition to the frontal eye, there are many other photoreceptors in the amphioxus CNS. Those developing in the hindbrain and spinal cord, called the organs of Hesse, are rhabdomeric or microvillar photoreceptors, while those in the forebrain, except for the Joseph cells, are ciliary photoreceptors. The organs of Hesse and the frontal eye consist of pigment cells containing melanin together with one or more photoreceptive neurons. However, the lamellar body, a ciliary photoreceptor, and the Joseph cells, which are rhabdomeric photoreceptors located in the posterior part of the cerebral vesicle, are not associated with pigment cells. The first photoreceptor to develop is an organ of Hesse in the middle of the hindbrain. The second is the frontal eye [20]. About the same time that the first organ of Hesse forms, at the early to mid-neurula, two rows of motor neurons also differentiate. Not surprisingly, the larvae are positively phototropic from the mid-neurula stage and begin muscular movements at the late neurula stage.

Gene expression is quite similar in the CNS of both amphioxus and vertebrates. Not only is the expression of genes involved in rostro-caudal patterning such as *Otx*, *Hox *and *Gbx *highly conserved [21-23], but also the expression of genes involved in the neuronal specification. For example, the *ERR*, *islet*, *Shox*, *Krox*, *Mnx *genes are expressed in developing motor neurons in both amphioxus and vertebrates [24-27]. However, it is not known whether expression of miRNAs is similar in the amphioxus and vertebrate CNS. In vertebrates, several miRNAs are expressed in specific brain regions or specific types of neurons suggesting their involvement both in development and function of the nervous system [8]. For example, in *Xenopus*, miR-124 is expressed in the optic vesicle and forebrain, where it negatively regulates *NeuroD1 *[9]. In the mouse brain, miR-124 seems to be largely restricted to differentiating and mature neurons [28], while miR-124 is expressed in neural progenitors [10,28]. Its expression appears to be controlled by a neuron restricted transcriptional repressor (REST/NRSF), which inhibits miR-124 expression in both nonneuronal cells and neural progenitors [29]. A second miRNA, miR-9, is also specifically expressed in proliferating neural precursors in both zebrafish embryos [30,31], and in mouse embryos and adults [28,32], while miR-7 and miR-184 are expressed in several types of brain tumors, suggesting a function in cell proliferation [33-36]. In addition, miR-137 has been implicated in proliferation of neural stem cells [37,38], while mir183 is expressed in hair cells in the vertebrate ear [39,40].

For amphioxus, there is only a single study of miRNA expression, which showed that two miRNAs (miR-133 and miR-1) are expressed in the developing muscular somites of amphioxus [13]. Therefore, to gain insights into the evolution and function of miRNAs during amphioxus neural development, we determined expression of six miRNAs (miR-124, miR-9, miR-7, miR-183, miR-184 and miR-137) in two species of amphioxus, *Branchiostoma floridae *and *B. lanceolatum*. All six miRNAs are known to be expressed exclusively or preferentially in the nervous systems of vertebrates and/or other species. Our results show that all six miRNAs are expressed in specific regions of the CNS of amphioxus, many of which can be correlated with cell types identified by microanatomical studies [20,41-43]. In addition miR-7, miR-137 and miR-184 are also expressed in endodermal and mesodermal tissues. Together with analysis of potential miRNA targets, these results give insights into the regulation of neurogenesis in amphioxus.

## Results

We found that all six miRNAs are expressed in the amphioxus CNS, although miR-7, miR-184 and miR-137 are also present in a strikingly broad spectrum of developing tissues derived from mesoderm and endoderm, while miR-183 is also expressed in some ectodermal sensory cells of the peripheral nervous system (PNS).

### miR-124 expression during amphioxus development

**Figure 1:**
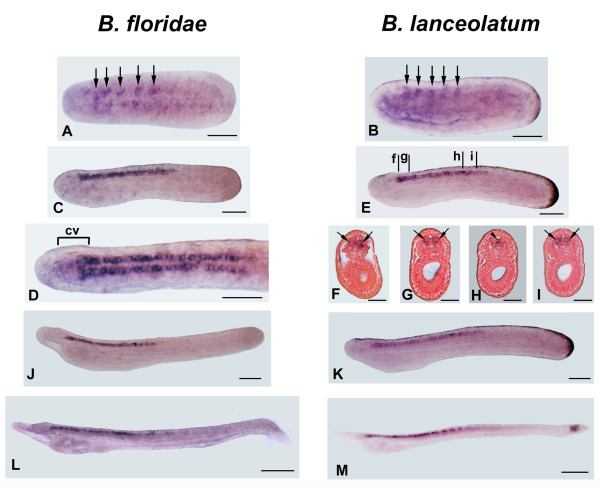
**Developmental expression of *miR-124 *in *B. floridae *(A-L) and *B. lanceolatum *(B-M) as analyzed by WM-LNA-ISH**. Anterior is to the left and dorsal is up in all whole mounts. Cross-sections are viewed from the anterior end of animal. Scale bars are 50 μm for whole mounts and 25 μm for transverse sections. **A, B**. Dorsal and side views of 14 h neurulae showing expression in five spots (arrows) in the neural plate. **C-E**. Side views of 22 hr neurulae. D: Detail in dorsal view of the specimen in C, showing the longitudinal rows of labeled nerve cells beginning at the posterior cerebral vesicle (cv) and extending to posterior neural tube. **F-I**. Transverse sections through levels in E show symmetrical ventrolateral labeled nerve cells (arrows) and a single cell in the ventromedial wall of the neural tube just near the first pigment spot of the dorsal ocellus (indicated by an arrowhead). **J, K**: Side views of early larva. **L, M**: Side views of first gill slit larvae. Dark granules in the tailbud region of the late-neurula and larval stages of *B. lanceolatum *are pigment and not a positive reaction for *miRNA *transcripts.

**Figure 2:**
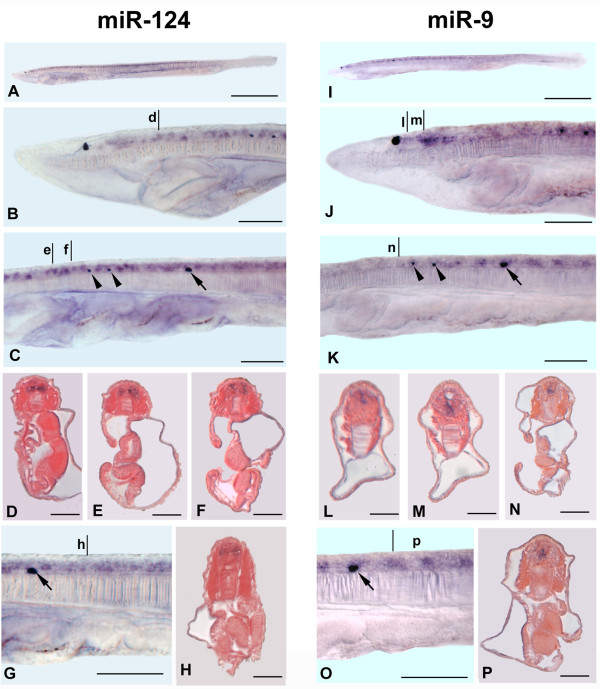
**Comparison between miR-124 and miR-9 expression at late larval stage of *B. floridae***. Anterior to the left. Cross section scale bars are 25 μm. **A, I: **Side views of larvae with three to four gill slits. Scale bars: 250 μm. **B, J**. Anterior enlargement of specimens in A and I, respectively. Scale bars: 50 μm. **C, K**. Enlarged view of the neural tube of specimen in A and I at level of three dorsal ocelli (the first dorsal ocellus appearing during the development and located near the somite 5 is shown by arrows, the most anterior ones are indicated by arrowheads. Each differing from the first dorsal ocellus in being bicellular associations between a single neuron and a single pigment cell). Scale bars: 50 μm. **D**: Cross section through level d in B. **E, F**: Cross sections through levels e and f in C. **L, M**: Cross section through levels l and m in J. **N**: Cross section through level n in K. **G, O**: Detail of specimens in A and I at levels of the first dorsal ocellus (arrows). Scale bars: 50 μm. **H, P**: Cross sections through levels h and p in G and O, respectively.

In both *B. floridae *and *B. lanceolatum*, the earliest miR-124 expression appears in 14 h mid-neurulae in a periodic pattern of cells on each side of the midline adjacent to the first five somites (Figure 1A, B). As development proceeds, expression is visible in two longitudinal rows of labeled nerve cells beginning at the posterior cerebral vesicle (cv) and extending to the hindbrain (Figure 1C, D). Plastic sections of the same embryos show that such cells are preferentially lateral and ventrolateral (Figure 1F-I) except for a few neurons in the floor plate and adjacent to the pigment cell of the first photoreceptor to form (Figures 1 H). In later larvae (three to four gill slits), expression of miRNA expands anteriorly into the cerebral vesicle, but does not reach the anteriormost portion at any stage examined (Figures 1 J-M and 2A, B). In particular, two areas of the cerebral vesicle express miR-124: one in the middle and the second in the posterior half of the cerebral vesicle (Figure 2B). Cross sections at different levels of the nervous system showed that most labeled cells are in the ventro-lateral neural tube (Figure 2D, E and 2H) whereas very few labeled cells were located in the dorsal wall (Figure 2F). Expression in the two rows of ventro-lateral cells also expands posteriorly into the spinal cord during larval development (Figure 2 A-H). This is in agreement with the pattern of neurogenin, which initially labels neurons anteriorly in the CNS with the pattern moving posteriorly as development proceeds.

### miR-9 expression in late larval stage of amphioxus *B. floridae*

miR-9 expression was never detected in embryos or very early larvae of both *B. floridae *and *B. lanceolatum*, in contrast to the situation in vertebrates [30,31]. Instead, miR-9 expression appears only later in larvae (three to four gill slits) (Figure 2I). Here, we show miR-9 expression exclusively in the *B. floridae*. miR-9 is expressed in scattered cells throughout the neural tube except at the most anterior part of the cerebral vesicle where the frontal eye has just formed (Figure 2J). Just posterior to the frontal eye, we observed miR-9 transcripts in a very few dorsal cells of the cerebral vesicle (Figure 2J, L). The miR-9 labelled cells cannot be the same as those expressing miR-124, because the former are organized as segmental blocks of cells (Figure 2K, O), while the latter, although segmentally arranged at an early stage, are no longer segmentally arranged in the larva (Figure 2B, C, G). Moreover, transverse sections clearly demonstrate that miR-9 expressing cells are mainly in the ventral midline of the neural tube (Figure 2M, N, P), while those of miR-124 are more lateral (Figure 2D-F, H).

### miR-7 expression in amphioxus

**Figure 3:**
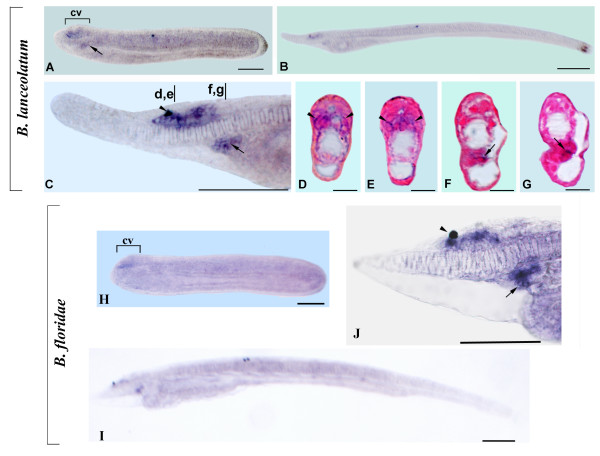
**miR-7 expression in *B. lanceolatum and B. floridae***. Anterior to the left and dorsal up in all whole mounts. Cross-sections are viewed from the anterior end of animal. Scale bars are 50 μm for whole mounts and 25 μm for cross sections. **A: **Side view of 22-h neurula. Expression is restricted to the cerebral vesicle (cv) and anterior pharyngeal endoderm (arrow). **B**: Side view of a first gill slit larva. **C**: Higher magnification of the anterior end of larva in **B**. Expression is observed in cerebral vesicle and preoral pit (arrow). **D, E**: Cross sections through levels d and e in C show several ventrolateral labeled cells of the anterior cerebral vesicle (arrowheads). **F, G**: Cross sections through levels f and g in C show miR-7 expression in cells of preoral pit (arrows). **H: **Side view of 20-h neurula showing expression in the cerebral vesicle. **I**: Side view of a three gill slit larva. **J**: Anterior enlargement of the anterior end of the specimen in I. miR-7 is expressed in cells of the frontal eye complex and in further regions of cerebral vesicle (cv). A strong signal was also found in the preoral pit (arrow). Arrowhead in C and J points to pigment spot associated with frontal eye.

Unlike miR-124 and miR-9, expression of miR-7 is not limited to the CNS. The first miR-7 expression appears at the late neurula stage (20 to 22 hours after fertilization) as a weak signal in the ventral portion of the cerebral vesicle (Figure 3A, J). At the late neural stage, miR-7 transcripts were also found in the most anterior end of the pharyngeal endoderm (Figure 3A) from which Hatschek's left anterior diverticulum, also known as the preoral organ or Hatschek's pit (a putative homolog to the vertebrate adenohypophysis), will originate. In the early larva, miR-7 is detectable in almost the entire ventral portion of the cerebral vesicle including the photoreceptors of the frontal eye (Figure 3B-E, 3I, J). Finally, a restricted expression was also observed in Hatchek's pit (Figure 3C, F, G, J).

### miR-183 expression in amphioxus larvae

**Figure 4:**
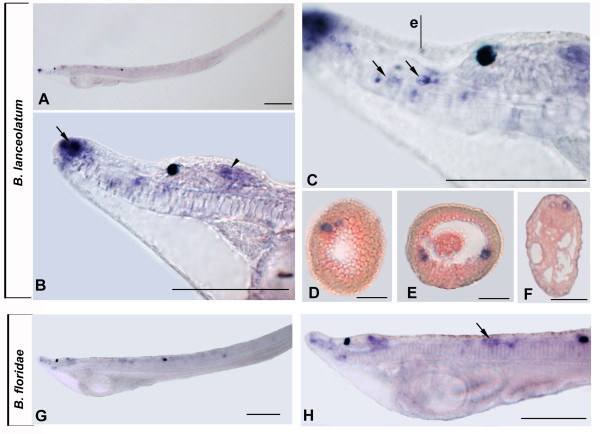
**miR-183 expression in *B. lanceolatum *and *B. floridae***. Anterior to the left and dorsal up in all whole mounts. Cross-sections are viewed from the anterior end of the animal. Scale bars are 50 μm for whole mounts and 25 μm for cross sections. **A: **Side view of a 72-h larva. **B: **Detail of the head region of larva in A showing expression in epidermal cells located in the most anterior tip of rostrum (arrow) and in dorsal wall of the posterior cerebral vesicle (arrowhead)**. C: **Higher magnification of specimen in A with in focus labeled epidermal cells on the sides of the rostrum (arrows). **D, F**: Cross sections through the levels indicated by an arrow (D) and an arrowhead (F) in B, respectively. **E**: Cross section through level e in C. **G**: Side view of *B. floridae *larva. **H**: Enlargement of the specimen in G showing expression in epidermal cells of rostrum, cells of the dorsal posterior region of cerebral vesicle and in scattered cells of the neural tube (arrow).

miR-183 expression is largely restricted to the CNS, but it is also expressed in some likely ectodermal sensory cells in the anterior end of the larva. The expression of miR-183 starts from the larval stage and it does not differ substantially between *B. lanceolatum *and *B. floridae *at 72 hr of development (Figure 4). Strong expression was detected in two groups of epidermal cells in the rostrum: the first is confined to the most anterior tip (Figure 4A, B, D, G, H), and the second is localized in the ectoderm anterior to the cerebral vesicle and adjacent to the notochord (Figure 4C, E, G, H), approximately where an anterior sense organ (the corpuscles of de Quatrefages) will later develop [44]. miR-183 was also found in some dorsolateral cells on either side of the posterior cerebral vesicle (Figure 4A, B, F-H). At least some of these cells are located approximately where the lamellar organ is developing. Finally, a few scattered cells expressing miR-183 were also found in the CNS posterior to the cerebral vesicle (Figure 4A, G, H).

### miR-184 expression in amphioxus

**Figure 5:**
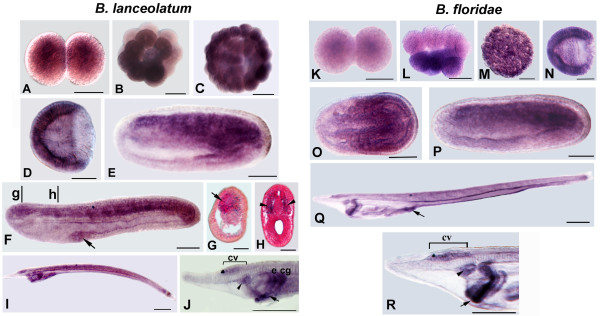
**miR-184 expression during *B. lanceolatum *and *B. floridae *development**. Anterior to the left and dorsal up in D, E, F, I, J. Scale bars are 50 μm for whole mounts and 25 μm for cross sections. **A-D, K-N**: 2-cell stage, 16-cell stage (B, viewed from the vegetative pole; L, side view), blastula stage (3.5 h post fertilization), cup-shaped gastrula (side views, 6 h and 7 h post fertilization in D and N, respectively). **O**: Dorsal view of early neurula with anterior toward left. **E, P**: Side view of mid-neurula. **F**: Side view of late neurula. Expression was found in the cerebral vesicle, somites and in the primordium of first gill slit (arrow). **G, H**: Cross sections through levels g and h in F showing expression in cells of the cerebral vesicle (arrow) and in somites (arrowheads). **I**: Side view of a first gill slit larva. **Q**: Side view of a two gill slit larva. **J, R**: Higher magnifications of anterior region of the specimen in I and R respectively showing expression in the cerebral vesicle (cv), preoral pit (arrowhead), endostyle (e) and club shaped gland (cg) (arrow).

mir-184 is expressed very early during development in both *B. floridae *and *B. lanceolatum *(Figure 5). It is ubiquitously expressed in two-cell embryos (Figure 5A, K). By the 16-cell stage, expression is limited to the vegetal blastomeres (Figure 5B, L), although in the early blastula, it is also expressed, albeit at a lower level, in the animal blastomeres (Figure 5C, M). At the gastrula stage, mir-184 is expressed throughout the presumptive neuroectoderm and at a higher level throughout the mesendoderm (Figure 5D, N). At the early (Figure 5O) and mid-neurula stages (Figure 5E, P), we detected expression in the entire neural tube, somites and posterior endoderm. This pattern is also maintained at the late neurula stage (Figure 5F-H), where miR-184 transcripts are evident in the cerebral vesicle (Figure 5G), somites (Figure 5H) and in a group of ventral endodermal cells from which will arise the first gill slit (Figure 5F). Finally, at the larval stage, transcripts of miR-184 are detectable in the anterior and intermediate regions of the cerebral vesicle, in the preoral pit, and in two pharyngeal organs (endostyle and club shaped gland) (Figure 5J, R) as well as in cells surrounding the first (Figure 5I) and second gill slits (Figure 5Q).

### miR-137 expression in amphioxus

**Figure 6:**
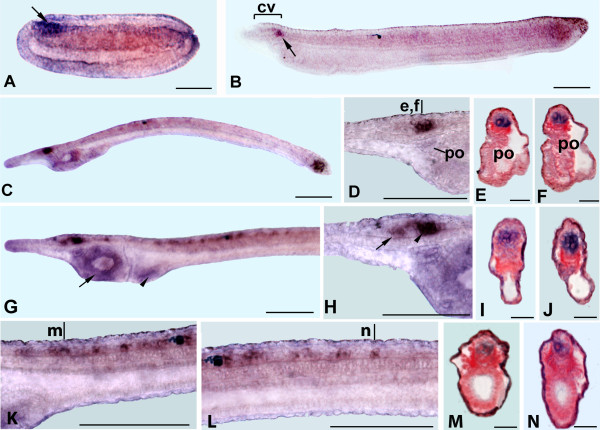
**miR-137 expression in *B. lanceolatum***. Anterior to the left and dorsal up in all whole mounts. Cross-sections are viewed from the anterior end of animal. Scale bars are 50 μm for whole mounts and 25 μm for cross sections. **A: **Side view of 16-h neurula. Expression was found in the most anterior part of the neural tube (arrow). **B**: Lateral view of 24 hr larva. Transcripts are visible in a cluster of cells (arrow) of the posterior cerebral vesicle (cv). **C**: Side view of 48-h larva. **D: **Higher magnification of the specimen in C with detail of labeled cells of the cerebral vesicle just above the preoral pit (po). **E, F: **Consecutive cross sections trough levels e and f in D showing transcripts in a group of ventrolateral cells that forms the infundibular organ. **G**: Side view of 72 hr larva. Expression was found in the neural tube and in cells located around the mouth (arrow) and in the first gill slit (arrowhead). **H**: Anterior enlargement of specimen in G. Two groups of cells in the cerebral vesicle express miR-137. The most anterior is found in the middle (arrow), the more posterior (arrowhead) correspond to the cells of the infundibular organ and to the tegmental neuropile. I: Cross section through level indicated by an arrow in H. J: Cross section through level indicated by an arrowhead in H, **K, L**: Enlargements of different parts of the neural tube of the specimen in G showing miR-137 expression in segmentally arranged nerve cells. **M, N**: cross sections through levels m and n in K and L, respectively. miR-137 is expressed in ventrolateral nerve cells.

**Figure 7:**
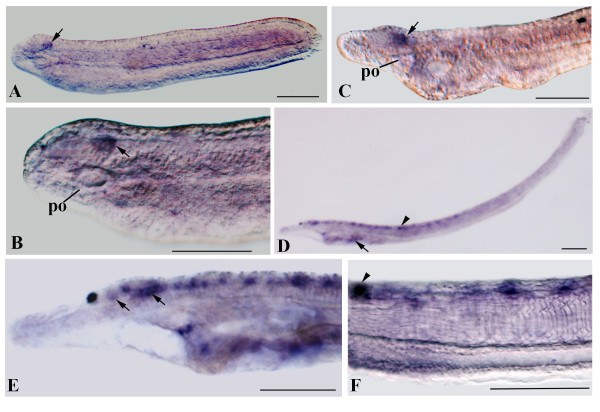
**miR-137 expression in *B. floridae***. **A: **Side view of 24-h larva. **B: **Anterior enlargement of the specimen in A showing expression in a discrete region of the posterior cerebral vesicle just above the preoral pit (po). **C: **Higher magnification of the anterior end of a 30-h larva. **D: **Side view of a three gill slit larva with expression in the neural tube and gill slits (arrow). **E: **Anterior of larva in D showing expression in different cells of cerebral vesicle (arrows) and in segmentally arranged cells of the neural tube just posterior. **F: **Enlargement of the posterior neural tube just posterior to the pigment of the first dorsal ocellus (arrowhead) with miR-137 transcripts in cells segmentally arranged in the neural tube.

miR-137 transcripts were first discernable during the neurula stage of *B. lanceolatum *(Figure 6) and *B. floridae *(data not shown). In the mid-neurula, expression was found exclusively in the most anterior end of the neural tube, the presumptive cerebral vesicle (Figure 6A). In the early larva (24 to 30 hours after fertilization), the signal was confined to a very few cells in the posterior cerebral vesicle (Figures 6B and 7A, B). From this stage onward, this signal gradually becomes more intense (Figures 6C, D and 7C). Transverse sections indicated that at least some of the labeled cells probably are in the presumptive infundibular organ (Figure 6E, F). However, miR-137 is likely also found just posterior to the infundibular organ in the tegmental neuropile. Subsequently, expression of miR-137 extended to an additional group of cells more anteriorly in the cerebral vesicle (Figures 6G, H and 7D, E) and also to cells arranged in a periodic pattern more posteriorly in the neural tube (Figures 6G, K-N and 7D-F). Non-neural expression was also found around the mouth and in the developing gill slits (Figures 6G and 7D, E)

### Amphioxus predicted miRNA targets

At present *in silico *prediction is generally the first approach that is used to identify potential miRNA targets. Table 1 and Additional file 1 (Tables S1 and S2) show that miR-124, miR-7, miR-183, miR-184 and miR-137 probably regulate different target genes, some of which could be regulated by many miRNAs. Comparison of the expression patterns of these miRNAs with those of their putative target mRNAs revealed both overlapping and non overlapping expression. Animal microRNA target prediction is particularly challenging because of the complexity of miRNA target recognition. At present, comprehensive rules controlling miRNA target recognition and binding have not been discovered, and false positive and false negative results are a problem. In animals, miRNA:mRNA duplexes often contain several mismatches, gaps and G:U base pairs in many positions, which limit the length of contiguous sequences of perfect nucleotide matching.

**Table 1:** Coexpression of miRNAs and enriched brain putative targets

putative miRNA targets	miR-124	miR-7	miR-183	miR-137	miR-184	References
**Calmodulin**	FT-RH-Mir		**FT-RH-Mir**		**FT-Pita-RH-Mir**	[109]

**Chordin**	**Pita-RH-Mir**					[110,52]

**Coe**					**FT-Pita-RH-Mir**	[111]

**Engrailed**					**FT-Pita-RH-Mir**	[112]

**FoxG (BF1)**	FT-Pita-RH-Mir				**FT-RH-Mir**	[113]

**Gbx**			FT-Pita-RH-Mir	FT-Pita-RH		[23]

**Hairy A**	FT-Pita-RH-Mir					[114]

**Mnx (2 sites)**					**FT-Pita-Mir**	[27]

					**FT-Pita-RH-Mir**	

**Netrin**			**FT-Pita-RH-Mir**			[115]

**NK2.2**		**FT-RH-Mir**				[116]

**Otx**					**FT-Pita-RH**	[21,52]

**Pax-2/5/8**			FT-Pita-RH			[117,52]

**Six-4/5**				**FT-RH-Mir**		[118]

**Snail**					**FT-RH-Mir**	[119]

**TH**					**FT-RH-Mir**	[120]

**TR2/4**				**FT-Pita-RH**	**FT-Pita-RH**	[121]

**Wnt8**	Pita-RH-Mir					[122]

**Expression of miRNA and enriched brain putative targets detected by at least three of the four bioinformatics algorithms (FindTar, Pita, RNAhybrid and miRanda)**. miRNA-target coexpression is written in bold. Abbreviations are the followings: FT, FindTar, RH, RNAhybrid, Mir, miRanda.

Often miRNAs inhibit translation of their direct targets, and, therefore, the miRNA and its target are co-expressed. However, miRNAs may also regulate the levels of expression of their target mRNAs. This mode of gene regulation is generally linked to a non overlapping expression between miRNAs and their targets [45-47]. However, there are also examples of partially or completely overlapping expression of targets and miRNAs [48]. Alternatively, the target can be expressed in cells that express the miRNA but at higher levels in those cells that do not express the miRNA [49,50]. Here, we used four different programs (miRanda, FindTar, RNAhybrid and PITA) to predict putative amphioxus miRNA targets. We argued that if at least three programs predict the same target sites, the likelihood of false positives is reduced [51]. Unfortunately, several common programs used for vertebrates, Drosophila and worms (ex. TargetScan and PicTar) are not available for "non standard" animal species such as amphioxus. Thus, we have erred on the side of caution and may have missed a number of targets. Indeed, almost one third of the predicted targets were not common to the data generated by three of the four methods.

Therefore, we searched 3'UTR sequences of 68 genes of *B. floridae *for potential binding sites of the six miRNAs (Additional file 1 Table S2). In particular, we selected some genes that are expressed in the nervous system during amphioxus development (Additional file 1 Table S2). For some of them expression in *B. lanceolatum *is also known [52] (Table 1). Of 68 genes expressed in the amphioxus nervous system, we found 17 transcripts with one or more sites for the 5 miRNAs (Table 1 and Additional file 1 Table S1). miR-9 does not show hits for the selected transcripts. Only eight miRNAs were predicted by at least 4 methods to bind at the same site. Our analysis shows that among 17 putative targets, expressed in the CNS of amphioxus, 13 are coexpressed with the one or more of the 5 miRNAs and 6 are not coexpressed (Table 1 and Additional File 1 Table S1). Therefore, we hypothesize that amphioxus miRNAs regulate targets both by controlling translation and by mRNA degradation.

Comparison of the expression patterns of the six miRNAs and their potential targets shows that only miR-184, which is very broadly expressed, is co-expressed with all the potential targets we identified (Table 1 and Additional file 1 Table S1). For example, in the CNS, miR-184 is co-expressed in the cerebral vesicle with calmodulin, FoxG (BF1), TH, TR2/4, Otx. In contrast, none of the selected transcripts contains target sites for miR-9. However, since there are relatively few data concerning gene expression at the late larval stage of amphioxus when miR-9 starts to be expressed, it is difficult to compare its expression with that of other potential targets.

The other 4 miRNAs are all co-expressed with one or more of their potential targets in the CNS. For example, among the several predicted targets of miR-124 expressed in the nervous system (Table 1 and Additional file 1 Table S1) only Chordin is co-expressed and only at the late neurula stage. At the larval stage, expression of the two no longer overlaps as chordin expression becomes restricted to the cerebral vesicle, where miR-124 is never expressed. Expression of FoxG (BF1), Hairy A, Wnt8 and Calmodulin never overlaps with that of miR-124. Transcripts of FoxG (BF1), Wnt8 and Calmodulin in the CNS are limited to the cerebral vesicle, while those of Hairy A in the CNS are posterior to that of miR-124, and by the larval stage become confined to endodermally-derived structures (Table 1).

The only putative target of miR-7 is NK2.2. In the CNS, expression of miR-7 and *NK2.2 *appears to overlap in the posterior 2/3 of the cerebral vesicle at the late neurula and larval stages. However, *NK2.2 *is also expressed in gut cells, while miR-7 is expressed in the preoral pit.

Among the predicted amphioxus targets of miR-183 we found two genes showing an overlapping expression: *Calmodulin *and *Netrin *(Table 1). For example, at larval stage *Netrin *is found in the lamellar body as mir-183. On the contrary, *Gbx *and *ax2/5/8 *are putative targets of miR-183 known to be expressed at larval stage in the hindbrain region where miR-183 is not present (Table 1).

Putative targets of amphioxus miR-137 are *Six-4/5, TR2/4/*and *Gbx *(Table 1). Of these, the first two are co-expressed with miR-137. *Six-4/5 *and TR2/4 are overlapping with miR-137 in some regions of cerebral vesicle and in cells of the mouth and gill slits, whereas *Gbx *is a putative target not coexpressed with miR-137.

## Discussion

**Figure 8:**
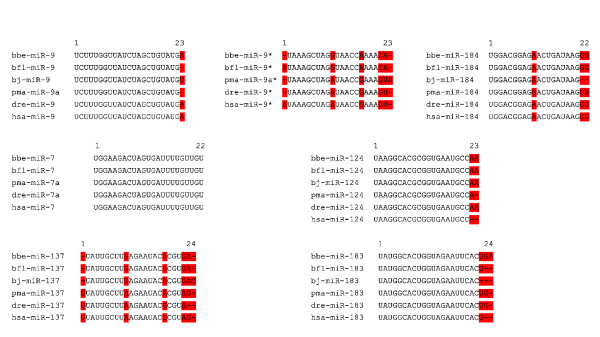
**Sequence alignment of mature miRNA sequences**. The alignment was performed using the mature miRNA sequences of three *Branchiostoma *species (*belcherii*, bbe; *floridae*, bfl; *japonicum*, bj) and three vertebrates (*Homo sapiens*, has; *Danio rerio*, dre; *Petromyzon marinus*, pma). Different residues and insertions/deletions are highlighted in red in the alignment. The miR-9* sequence alignment showed several differences between *Branchiostoma sp. a*nd vertebrates. The sequences of mature miRNAs of *B. floridae, Homo sapiens, Danio rerio, Petromyzon marinus *were obtained from miRBase. The sequences of miRNAs of *B. belcherii *and B. *japonicum *from [12,13].

Cephalochordates, commonly known as amphioxus or lancelets, are the most basal, living group of chordates. At least 20 species of the genus *Branchiostoma *have been described but only three of them are mainly investigated: *B. floridae *in North America, *B. lanceolatum *in Europe, and *B. belcheri *in East Asia. Mitochondrial DNA sequences indicate that they probably diverged from 100 to 200 Mya [53,54]. These species are morphologically very much the same. For *B. lanceolatum *and *B. floridae*, the major differences in larvae are the long anterior end and the pigmented tail in the former. Given this high level of morphological conservation, it is not surprising that developmental gene expression is proving to be nearly identical in the two species [52]. Thus, it is not also surprising that miRNAs are conserved between *B. floridae*, *B. belcheri *and *B. japonicum *[11-13] (Figure 8) and that we found their expression patterns in at least *B. floridae *and *B. lanceolatum *also to be much the same. For the six mature miRNAs sequences investigated in the present study, there are a few differences in sequences including insertion/deletion of few nucleotides in the 3' ends of miR-9, miR-184, miR-183 and miR-137 (Figure 8), outside the 5'-seed region (two to eight nucleotides), which is known to be involved in target recognition. In fact, there is also a high level of sequence identity among these miRNAs in vertebrates and *Branchiostoma*. During miRNA biogenesis, one strand of the RNA duplex is preferentially selected for entry into a silencing complex, whereas the other strand, known as the passenger strand or miRNA* strand, is degraded. Recently, some miRNA* sequences were reported to be abundantly expressed, and generally are less conserved across species. This situation is particularly evident for miR-9* with high levels of nucleotide divergence despite of their partners were highly conserved (Figure 8).

### Amphioxus miRNAs show spatially localized expression in CNS

**Figure 9:**
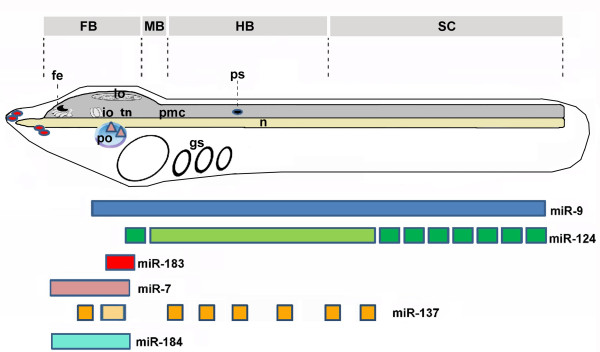
**Schematic representation of the left side of amphioxus larva showing the expression of miRNAs in CNS and PNS**. Above the figure the putative territories homologous to the vertebrate diencephalon (D), midbrain (M), hindbrain (HB) and spinal cord (SC) are reported. The expression of miRNAs are shown by colored boxes. Different color shades of boxes indicate different stages of development in which we observed the appearance of new expression domains (light green correspond to the expression of miR-124 up to larva with a first gill and dark green miR-124 expression at larva with 3-4 gill slits; light yellow indicates miR-137 expression until 48 hour larva and dark yellow miR-137 expression at 72 hour larva). miR-7 and miR-183 transcripts in the preoral organ and epidermal sensory cells of PNS (red spots and pink triangles respectively) are also represented. The position of the frontal eye complex (fe), infundibular cells (io), tegmental neuropile (tg), lamellar organ (lb) and primary motor centre (pmc) are indicated. The first pigment spot of dorsal ocellus is also indicate (ps) as well as preoral organ (po), gill slits (gs) and notochord (n).

The CNS of larval amphioxus comprises an anterior cerebral vesicle (diencephalon plus small midbrain) and a long nerve cord (hindbrain plus spinal cord). The cerebral vesicle, includes the frontal eye rostrally, the infundibular organ ventrally and the lamellar organ dorsocaudally, which is also a photoreceptor. Just caudal to the infundibular organ, there is a thick ventral commissure (tegmental neuropile) receiving fibers that originate from the lamellate cells and just behind this is the primary motor centre (pmc) containing the anteriormost motoneurons and large premotor interneurons with caudal projections (Figure 9). The latter represents the ventral control locomotory system of amphioxus larva thought to be involved in initiating swimming and escape behaviors [18,19,55]. According to the microanatomical reconstructions and gene expression data, the posterior neural tube of amphioxus does not have segmented rhombomeres as vertebrates; but the collinear expression pattern of *AmphiHox*, as well as the segmental expression pattern of *islet *in the amphioxus neural tube, suggests that amphioxus have at least a hindbrain-related region [25,56] (Figure 9).

Our study shows that miRNAs in amphioxus are differentially expressed within the CNS suggesting that they have region-specific functions. Within the cerebral vesicle, miR-137 and miR-183 show restricted expression in specific cell types, whereas miR-184 and miR-7 are broadly expressed (Figure 9). In 48 h larvae, miR-137 is expressed ventrally in the infundibular organ and likely more posterior in the tegmental neuropile, whereas miR-183 is found dorsal to the infundibulum. At the same stage, miR-124 is preferentially expressed in the hindbrain, whereas in the cerebral vesicle it appears to be limited to few cells in the more posterior part (Figure 9). Moreover, miR-124 expression is also probably found in both interneurons and motoneurons located ventro-laterally in the pmc. Regional expression of miR-9 is similar to that of miR-124, although the exact cells in the neural tube expressing the two may not be the same. The regionally restricted expression suggests that miRNAs may regulate specific cell types in the CNS of amphioxus. These miRNAs could act in the regionalization of the nervous system in a manner similar to that of miR-9 in the positioning of the midbrain-hindbrain boundary (MHB) in zebrafish [31]. In addition, expression of some amphioxus miRNAs suggested roles in neuronal specification and/or differentiation. For instance, miR-124 is initially expressed where five pairs of motor neurons develop in the hindbrain. This segmental pattern is very similar to that of several genes including *islet*, *Krox*, *Mnx*, *ERR*, *Shox*, *synapsin *and *ChAT/VAChT *[24-27,57,58]. However, neither of these genes seems to be recognized by miR-124.

### Conservation of miRNAs across species

Given the sequence conservation of many miRNAs across species (Figure 8), it is perhaps not surprising that their developmental roles seem to be quite conserved as well. For example, miR-124 is a highly conserved miRNA expressed in the nervous systems not only of amphioxus and vertebrates, but also of several invertebrates including some lophotrochozoans and ecdysozoans, but not in *C. elegans *and *Aplysia*, where it is mostly confined to sensory neurons in the PNS [28,30,59-65]. In mammals, miR-124 is the most abundant miRNA in the embryonic and adult brain [60]. In particular, during neurogenesis, miR-124 is present at undetectable or very low levels in neural progenitors but is highly expressed in differentiating and mature neurons [10,28,66,67]. In amphioxus, miR-124 is expressed exclusively in the CNS, initially, in five groups of cells located ventro-laterally and ventrally in the midbrain and hindbrain at mid-neurula stage and later, more anteriorly, but still mostly in ventro-lateral neurons. Early expression of *Neurogenin *and *ERR *in the first motor neurons to differentiate is similar to that of miR-124 [24,68]. However, whether miR-124 is expressed in the same or adjacent cells is not certain. Certainly, more cells express miR-124 than expresses *ERR*.

In vertebrates, miR-124 is expressed in proliferating neuroblasts. However, in amphioxus, at least at the mid-neurula stage, expression of miR-124 seems to be in post-mitotic cells. At that stage, the only proliferating cells are in the most anterior and posterior-third of the neural tube [69] where miR-124 expression is not found. Interestingly, miR-124 expression was found in the most posterior part of the nerve cord only in later larvae, probably because of the delayed differentiation of neurons there.

miR-9 and miR-124 are both widely expressed in the amphioxus CNS, however cross-sections show that they are expressed in different cells with miR-124 being largely expressed ventrolaterally whereas miR-9 is expressed in the ventral midline. Moreover, unlike mir-124, miR-9 expression is limited to later larvae and it is likely not involved in neurogenesis as described in mammals. In addition the amphioxus miR-9* sequence in amphioxus has little sequence identity with its vertebrate counterparts (Figure 8), and like miR-9 it is probably not involved in the proliferation of neuronal progenitors.

Although amphioxus and vertebrate miR-9 genes are both expressed in the brain [60,70], unlike miR-124, the functions of miR-9 do not appear to be highly evolutionarily conserved across phyla. In zebrafish and mice, miR-9 is expressed in neural progenitor cells and promotes neurogenesis by repressing suppressors of neuronal differentiation. In addition, miR-9 also targets members of the transcriptional repressor REST complex, inhibiting the expression of neuronal genes in non-neuronal cells [29,71]. Several studies have suggested that miR-9 and miR-9* can both suppress progenitor proliferation and promote neural differentiation [66,71-73]. In zebrafish, miR-9 helps establish the MHB by antagonizing FGF signaling, thus, promoting neurogenesis [31]. However, miR-9 is also expressed in differentiated neurons of the telencephalon [10,66,74]. Moreover, miR-9 is one of four highest expressed miRNA genes in the adult brain of the lamprey *Petromyzon *marinus [75]. In contrast, in *Drosophila *embryos, miR-9 is not expressed in the CNS. Instead, it is expressed in ectodermal cells near some sensory organ precursors (SOPs), where it regulates the specification of sensory neurons by repressing the proneural gene *senseless *in the ectodermal non-SOP cells [76]. The difference between miR-9 function in *Drosophila *and vertebrates might be because miR-9 regulates different genes in these two organisms -- for example, proneural genes in *Drosophila *and inhibitors of neurogenesis in vertebrates. In other protostomes, such as the annelid *Platynereis dumerilii*, miR-9 is expressed in cells at the base of the antennae, a pair of head appendages likely acting as chemosensory organs [59].

In amphioxus, miR-184 is ubiquitously expressed in early embryos and larvae although there is expression in the cerebral vesicle in later larvae (Figure 9). In other species, it is similarly widely expressed. For example, it is expressed not only in the CNS, but also in eye and testis of mouse, pharyngeal arch ectoderm and lateral plate mesoderm in the chick, and in the developing eye, hatching gland and epidermis in the zebrafish [30,77]. Similarly in the lamprey, miR-184 is expressed in several organs (brain, gills, kidney, heart, muscle) [75]. Moreover, in *Drosophila*, while miR-184 transcripts are present at high levels in the CNS, they are also present at high levels in eggs and during early embryogenesis in the mesoderm and anterior endoderm as well as in the female germline [63,78,79].

Expression of miR-7 is comparatively evolutionarily conserved. In amphioxus, miR-7 expression in the CNS is limited to the cerebral vesicle (Figure 9). In late neurulae, it is expressed in the presumptive frontal eye, which consists of a cluster of pigment cells (the pigmented cup) and four rows of neurons [20] and where serotonin-containing cells will later differentiate [80]. In larvae, miR-7 transcripts also occur in the middle and posterior cerebral vesicle, including the infundibular cells. Interestingly, we also found miR-7 expression in developing preoral pit in amphioxus (Figure 9), which has been suggested to be homologous to the vertebrate pituitary [81-83].

In other species, miR-7 is also expressed in photoreceptors [84]. In *Drosophila*, together with repression of Yan protein (a repressor of retinal cell differentiation), miR-7 regulates photoreceptor differentiation induced by FGF signaling [85]. In the mouse, and zebrafish miR-7 is also expressed in the hypothalamus in neurons with sensory or neurosecretory functions [86,87]. Similarly, in the annelid *Platynereis*, miR-7 is expressed in vasotocinergic, FMRFamidergic and serotonergic neurons. In addition, miR-7 is expressed in some non-neural tissues including endocrine cells of the pancreas (islets of Langerhans) [88] and in the pituitary gland in mammals [77]. In lamprey, miR-7 is expressed at the larval and adult stage. The brain is the major site of production of such miRNA, although other organs (kidney, skin and gills) express it at low levels [75].

Expression of miR-137 is also highly conserved across species. Vertebrate miR-137, like miR-7, is expressed in specific nuclei in the thalamus/hypothalamus and dorsal tegmentum [66] and enriched in the synaptic compartment of neurons in the mammalian forebrain [89,90]. In the annelid *Platynereis*, miR-137 and miR-7 are expressed in the same region of the CNS [59], while in amphioxus, miR-137 starts to be expressed in some cells of the infundibular organ and in the tegmental neuropile (Figure 9) [19,91]. Moreover, in later larvae it is also expressed in other regions of the cerebral vesicle and in the posterior neural tube. Outside the neural tube we also found expression in the mouth region and gill slits (Figures 7 and 8). Similarly, in the lamprey miR-137 is also expressed in the mouth as well as in the brain [75].

### miR-183 family members represent one of the most highly conserved regulators in sensory organ development

In vertebrates, miR-183 family members (miR-182, miR-96, and miR-183) are co-transcribed from a 1 to 4 kb segment of intergenic DNA [92] and miR-183 and miR-96 genes appear to be adjacent in genomes of the urochordate *Ciona intestinalis *[93]. More recently, the genomic linkage of miR-182/miR-9/miR-183 has been described in other deuterostomes (*Strongylocentrotus purpuratus, Saccoglossus kowalevskii and Branchiostoma floridae*) [13]. Expression of miR-183 in sensory cells is highly conserved. In amphioxus, it is expressed in potential precursors of ectodermal sensory cells in the rostrum (Figure 9) [44] and, where the lamellar body, thought homologous to the epiphysis of agnathans [41], which also expresses miR-183 [30]. In vertebrates, the three miR 183 family members have very similar expression patterns in specific sensory cell types in the eye, nose, and inner ear [30,40,92]. miR-183 expression is also conserved in neuromasts of teleosts, amphibians and agnathans [93]. In mammals, the miR-183 family was predicted to recognize several genes known to have important roles in various sensory organs, supporting the hypothesis of the sensory organ specificity of this cluster [92]. In *Drosophila *and *C. elegans*, miR-183 homologs are also expressed in ciliated sensory cells of mechano- and chemo-sensory organs [93,94]. Similarly, in the hemichordate *Saccoglossus kowalevskii*, several potential neurosensory cells express miR-183 [93].

## Conclusion

Many miRNAs are evolutionarily conserved and some have limited expression patterns with strict tissue, cell, and temporal specificities, while others demonstrate ubiquitous or constitutive expression. Moreover, the high phylogenetic conservation of several miRNAs suggests their ancient origin and crucial function in evolutionarily conserved developmental processes.

Our study has set the stage for future investigations of control of neuronal development by microRNA in amphioxus. Amphioxus is a particularly favorable model given the small number of neurons in this model chordate and previous studies mapping each neuron and its connections in the amphioxus brain. In light of the conservation of neural patterning and miRNA expression in amphioxus and vertebrates, results from amphioxus should be highly relevant to understanding the mechanisms of neuronal patterning in the more complex vertebrate brain.

## Methods

### Embryos collection and whole-mount *in situ *hybridisation

Adults amphioxus (*Branchiostoma floridae*) were collected from Old Tampa Bay, FL, USA. *In vitro *fertilization, embryo culture, and fixation were performed as described [95]. Adults amphioxus (*Branchiostoma lanceolatum*) were collected in the bay of Argelès-sur-Mer, France, and gametes were obtained by heat stimulation [96,97]. Fixation was performed as described [95]. Expression patterns of mature miRNAs were determined by whole-mount *in situ *hybridizations using Locked Nucleic Acid (LNA)-modified oligonucleotide probes (WM-LNA-ISH). The sequences of mature miRNAs of *B. floridae *were obtained from miRBase release 17 [98]. LNA-modified probes were purchased from Exiqon (Denmark) and were directly labeled with DIG at both their 5' and 3'-ends, or at 3'-ends only, as previously described [10]. The sequences of LNA probes are the following: miR-124: 5'-GGCATTCACCGCGTGCCTTA-3'; miR-9: 5'-CATACAGCTAGATAACCAAAGA-3'; miR-7: 5'-ACAACAAAATCACTAGTCTTCCA-3'; miR-183: 5'-AGTGAATTCTACCAGTGCCATA-3'; miR-184: 5'-CCCTTATCAGTTCTCCGTCCA-3'; miR-137: 5'-ACGTGTATTCTCAAGCAAT-3'. Whole mount *in situ *hybridization was performed essentially as described by Holland and coworkers [95] with some modifications. In particular, the temperature of hybridization and subsequent washing steps was adjusted to approximately 22°C below the predicted melting temperatures of the LNA modified probes. All miRNA probes were used at the same concentration (final concentration 5 nM) and in parallel. To determine the miRNA expression patterns more precisely at the cellular level, some embryos were also sectioned. Labeled embryos were counterstained with 1% Ponceau S in 1% acetic acid, dehydrated in ethanol, embedded in Spurr's resin, and sectioned at 3 to 4 μm. Images were collected by using an Olympus IX71 microscope (Olympus Italia s.r.l., Italy) equipped with the chilled color digital camera ColorView II (Soft Imaging System GmbH, Germany) and images were processed using the analySIS software package (Soft Imaging System GmbH, Germany).

### Prediction of amphioxus microRNA target genes

To identify potential target genes of the six miRNAs analyzed in the present paper we examined a suite of RNAs known to be expressed in the nervous system (Additional file 1 Table S2). Because the *B. floridae *genome is not fully annotated, and several transcripts did not contain the 5' UTR and 3' UTR regions we selected full-length cDNA sequences to allow identification of the stop codons and consequently the 3'UTRs. Therefore, sequences of 3'UTRs were obtained from the *B. floridae *EST data sets [99] and from the JGI web site [100]. To improve the accuracy of target prediction we used four target prediction algorithms: miRanda, RNAhybrid, FindTar, PITA [101-104]. miRNAs with binding sites in the 3'-UTR as predicted by three of the four bioinformatics algorithms were selected as putative regulators of transcripts (Table 1 and Additional file 1 Table S1). For target prediction with miRanda software we used the following parameters: gap opening penalty of -8; a gap extension penalty of -2; a score threshold of 60; an energy threshold of -20 kcal/mol; a scaling parameter of 2. For RNAhybrid we considered only the sites with ΔG < -20 kcal/mol, with seed 2 to 8 and allowing G:U wobble pairs. PITA identifies putative targets assigning an energetic score (ddG). Only sites with ddG values below -10 were considered as significant. For FindTar we used default parameters: loop score > 15 and ΔG < -15 kcal/mol. PITA, FindTar and miRanda executable can be downloaded from their specific websites [105-107]. RNAhybrid is available at BIBiserv [108].

## Abbreviations

miRNA: microRNA; UTRs: untranslated regions; CNS: central nervous system; *B. floridae *and *B-lanceolatum*: *Branchiostoma floridae *and *Branchiostoma lanceolatum*; PNS: peripheral nervous system; REST/NRSF: RE1 silencing transcription factor/neural-restrictive silencing factor; pmc: primary motor centre; MHB: midbrain-hindbrain boundary; SOPs: sensory organ precursors; WM-LNA-ISH: whole-mount *in situ *hybridizations using Locked nucleic acid (LNA)-modified oligonucleotide probes.

## Competing interests

The authors declare that they have no competing interests.

## Authors' contributions

SC carried out the bioinformatic and *in situ *hybridization assays and drafted the manuscript. LM and GG contributed to the whole mount *in situ *experiments and bioinformatic analysis, respectively. DDPT was involved in the comparison of the miRNA and expression patterns and contributed to data discussion. SC and MP were responsible for the cellular and evolutionary interpretation of the data. All authors read and approved the final manuscript.

## Supplementary Material

Additional file 1 **Amphioxus putative miRNA targets**. Additional file 1, Table S1: Amphioxus putative miRNA targets expressed in the nervous system. Score values, energetic scores (ddG), free energy (ΔG) and loop scores produced by the different target prediction tools are shown. Additional file 1, **Table S2**: Complete list of transcripts analyzed by miRNA target prediction programs. Click here for file
